# Microbial Nanotechnology for Bioremediation of Industrial Wastewater

**DOI:** 10.3389/fmicb.2020.590631

**Published:** 2020-11-02

**Authors:** Mandeep, Pratyoosh Shukla

**Affiliations:** Enzyme Technology and Protein Bioinformatics Laboratory, Department of Microbiology, Maharshi Dayanand University, Rohtak, India

**Keywords:** microbial nanotechnology, nanoparticles, bioremediation, carbon nanotubes, industrial effluents, green technology

## Abstract

Pollutant removal from industrial effluents is a big challenge for industries. These pollutants pose a great risk to the environment. Nanotechnology can reduce the expenditure made by industries to mitigate these pollutants through the production of eco-friendly nanomaterials. Nanomaterials are gaining attention due to their enhanced physical, chemical, and mechanical properties. Using microorganisms in the production of nanoparticles provides an even greater boost to green biotechnology as an emerging field of nanotechnology for sustainable production and cost reduction. In this mini review, efforts are made to discuss the various aspects of industrial effluent bioremediation through microbial nanotechnology integration. The use of enzymes with nanotechnology has produced higher activity and reusability of enzymes. This mini review also provides an insight into the advantages of the use of nanotechnology as compared to conventional practices in these areas.

## Introduction

Water is essential for the continuation of life on earth, so the removal of pollution from water is just as crucial. Industrialization has put immense pressure on water use due to its use in production. Increased production leads to the generation of a huge amount of industrial effluents. The treatment of these industrial effluents is required in a strict and cost-effective way for the sustainable development of industries and the environment. Various electrochemical, advanced oxidation processes, and valorization techniques have been applied to reduce the toxicity of effluents from wastewater and for making its use sustainable (Gupta and Shukla, 2020). But these techniques are not cost effective for all industries. The development of nanotechnology and nanoscience has opened new avenues for the remediation of water pollutants. The nanotechnological pathways are more efficient than their conventional counterparts due to their smaller size, high surface area to volume ratio, and superior chemical properties (Baruah et al., 2019). Synthesis of green nanomaterials from microorganisms and extracts of other organisms have paved a path toward the eco-friendly remediation of pollutants. Iron nanoparticles are green nanoparticles which are used in remediation due to their redox potential while reacting with water, magnetic susceptibility, and non-toxic nature (Bolade et al., 2020).

Membrane-associated nanomaterials are also an effective method for effluent removal. Nanomaterials improve membrane permeability, stinking resistance, mechanical and temperature strength, and present innovative functions for pollutant degradation. Nano-catalysts also play a major role in the enhancement of degradation reactions (Corsi et al., 2018). Apart from membranes and nano-catalysts, metal-organic frameworks (MOFs) are employed for the removal of heavy metals from wastewater. These MOFs are synthesized by the coordination of organic ligands with metal ion precursors. MOFs can be made more effective by the coordination of functional groups with metal as opposed to the organic ligand. This is because of the less steric hindrance of metals (Deshpande et al., 2020).

In this mini review, we will discuss the use of such nanoparticles for the removal of pollutants in industries. Also, the use of microorganisms and enzyme-assisted green nanotechnology to remove and valorize waste materials is discussed.

## Nanotechnology in Wastewater Treatment

**FIGURE 1:**
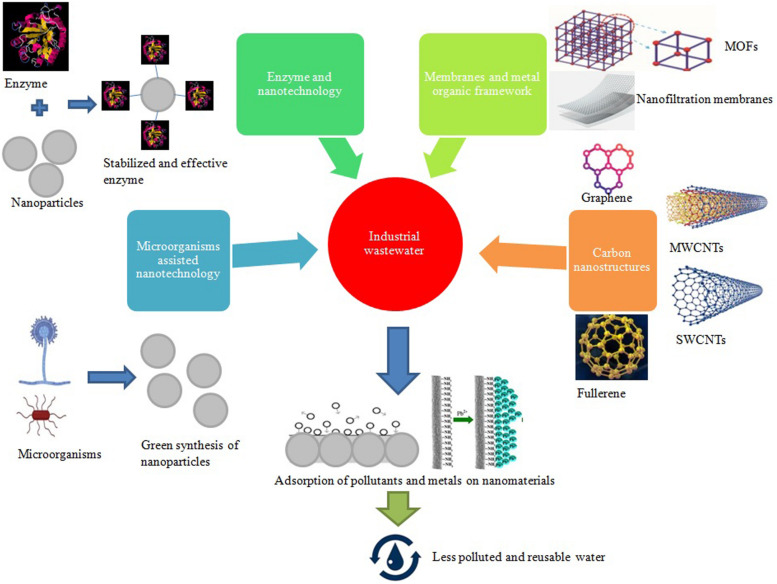
Utilization of various nanotechnology approaches in combination with microbial assistance for wastewater bioremediation. MOFs: Metal organic frameworks; MWCNTs: multi-walled carbon nanotubes; SWCNTs: Single-walled carbon nanotubes.

The smaller size of nanomaterials makes them suitable for use in the treatment of wastewater. They have specific chemical, physical, and biological properties that enhance their use in various applications. Different nanomaterials, such as carbon-based (Nanocomposites or Nanotubes), metals and their oxides-based nanomaterials, have been used for effluent removal from wastewater. Wastewater management practices consist of photocatalytic degradation, adsorption, filtration through nanoparticles, and observation of different contaminants and pollutants (Palit and Hussain, 2020). Figure 1 shows the use of various nano-techniques applied for bioremediation of industrial effluents.

### Nano-Adsorbents and Nanofiltration Membranes

Nanoparticles have been widely used as adsorbents to remove harmful contaminants from industrial wastewater. Nano-adsorbents can remove organic and inorganic pollutants (Kumari et al., 2019). They are categorized mainly as carbon-based, metal, and metal oxide-based nanoparticles. Carbon-based nanoparticles mainly include carbon nanotubes (CNTs), activated carbon, graphene, and, to some extent, fullerene (Kumari et al., 2019). Carbon nanotubes act as adsorbents for toxic chemicals from manufacturing industries or pharmaceutical wastewater. Kariim et al. (2020) prepared multi-walled carbon nanotubes (MWCNTs) from Fe-Ni supported on activated carbon using the chemical vapor deposition (CVD) technique. Adsorption characteristics of 2.5961 and 2.1363 were found for metronidazole and levofloxacin, respectively, which come in a range of good adsorption of 2–10. The enthalpy change showed that metronidazole was chemisorbed and levofloxacin was physisorbed. Similarly, MWCNTs are also able to adsorb metals from wastewater. The carboxylated MWCNTs were able to achieve increased adsorption of As(V) and Mn(VII) at 250 and 298 mg/g, respectively. The thermodynamic results showed metal removal takes place through chemisorptions (Egbosiuba et al., 2020).

Activated carbon modified nano-magnets have also been used for the removal of fluoride ions from wastewater. The nanocomposite was able to remove 97.4% fluoride ion from synthetic wastewater by sorption with an uptake of 454.54 mg/g (Takmil et al., 2020). Recently, microbial fuel cells and nanocatalysts are also used for the generation of bioelectricity. Electrodes coated with Iron(II)molybdate nanocomposites were able to enhance the efficiency of microbial fuel cells. Using this method, the maximum columbic efficiency of 21.3 ± 0.5%, the power density of 106 ± 3 mW/m^2,^ and COD removal competence of 79.8 ± 1.5% was achieved (Mohamed et al., 2020). Superparamagnetic composite of iron oxide nanoparticles with activated carbon was found to be suitable to remove Cr(VI) from wastewater. The treated wastewater was suitable for discharge according to the environmental protection agency (EPA)’s recommendations. Magnetic separation and sorption were utilized for the removal of heavy metal (Nogueira et al., 2019). A graphene-based nanocollector was prepared by Hoseinian et al. (2020). They prepared an amino-functionalized graphene oxide nanocollector to remove nickel ions by using an ion floatation process. They were able to achieve around 100% removal of nickel ions from the wastewater using this economical, efficient, and stable nanocollector (Hoseinian et al., 2020).

Metal and metal oxide-based nano-adsorbents also play a vital role in the removal of pollutants from wastewater. Studies have revealed that coating magnetic nanoparticles with other supports led to an increase in their adsorption efficiency. Magnetic nanoparticles coated with silver showed 36.56% chemical oxygen demand (COD) removal from wastewater, which was 6.16% higher than the uncoated magnetic nanoparticles (Najafpoor et al., 2020). Similarly, a magnetic polymer of Co_3_O_4_@SiO_2_ magnetic nanoparticles coated with nylon 6 was able to adsorb 666.67 mg/g Pb (II) from wastewater at 298K. The polymer was reusable up to six cycles with a little loss in its adsorption capacity of < 30% (Mohammadi et al., 2020). Thus, these nanoparticles, alone as well as in conjugation with other favorable supports, can remove pollutants from industrial effluents. Their reusability and stability make them cost effective and environmentally friendly. Similarly, metal oxide-based nanomaterials work efficiently in effluent treatment. Nano-porous magnesium oxide obtained from the solid waste from the ductile iron industry was able to adsorb 1,000 mg.g^–1^ of toxic dye from the wastewater. The adsorbent properties were enhanced by using a 1:1 mixture of sodium dodecyl sulfate (SDS) and polyoxyethyleneoctyl phenyl ether (TX100). This helped to achieve a suitable pore size of 16 nm (Pourrahim et al., 2020). Almomani et al. (2020) used magnetic nanoparticles, made from iron oxide, to remove heavy metals from wastewater. The iron oxide nanoparticles grafted on hyperbranched polyglycerol were able to remove nickel, copper, and aluminum from wastewater in 35 s. Moreover, the organic content and phosphorous do not affect the adsorption efficiency of nanoparticles, while nitrogen reduced the removal of heavy metals.

Nanofiltration (NF) membranes also play a vital role in the recovery of nutrients from industrial effluents. NF90 gives the highest rejection (70%) of phosphorous from pulp and paper industry effluent. But the problem in the fouling of membrane arises due to the high phosphorous content (Leo et al., 2011). Shalaby et al. (2020) used gold nanoparticles woven with a polymer blend of the NF membrane to achieve a higher recovery of phosphorous from wastewater. They were able to achieve 96.1% rejection of trivalent phosphate with increased fouling resistance and hydrophobicity of the membrane (Shalaby et al., 2020). In another study, Ceramic supported graphene oxide (GO)/Attapulgite (ATP) composite membrane was prepared for the removal of heavy metals. The membrane was able to reject nearly 100% of metal ions of copper, nickel, lead, and cadmium. It is valuable to prepare such a membrane due to its increased flux speed, immense water stability, and outstanding rejection ability (Liu et al., 2019). There are some concerns related to the chemical nature of nanomaterials with commercialization. So, researchers have now utilized microorganisms for the generation of green nanostructures. These microorganisms-supported nanotechnological applications have led to a revolution in the field of green nanotechnology.

## Microorganisms Assisted Nanotechnology

Biofabrication of nanomaterials and the simultaneous use of microbes makes the use of nanotechnology more sustainable and eco-friendly. The chemically produced nanoparticles may have some disadvantages in relation to the use of chemicals and self-agglomeration in aqueous solution. So, the green synthesis of nanoparticles from plant extract, fungal, and bacterial enzymes can be a potential solution. They act as reductive agents for the metal complex salt and generate metallic nanoparticles. These nanoparticles attain superior solidity in an aqueous environment because of co-precipitation or by adding proteinaceous and bioactive elements onto the outer face of the nanoparticles. Mahanty et al. (2020) biofabricated iron oxide nanoparticles from *Aspergillus tubingensis* (STSP 25) obtained from the rhizosphere of *Avicennia officinalis* in Sundarbans, India. The synthesized nanoparticles were able to remove more than 90% of heavy metals [Pb (II), Ni (II), Cu (II), and Zn (II)] from wastewater with a regeneration capability of up to five cycles. The metal ions were chemically adsorbed on the surface of the nanoparticles in endothermic reactions (Mahanty et al., 2020). In another study, exopolysaccharides (EPS) obtained from *Chlorella vulgaris* were used to co-precipitate with iron oxide nanoparticles. The Fourier-transform infrared spectroscopy (FT-IR) analysis revealed the successful modification of nanoparticles by functional groups of EPS. Further, it was observed that the nanocomposite was able to remove 91% of PO_4_^3–^ and 85% of NH_4_^+^ (Govarthanan et al., 2020).

The synthesis of nanoparticles with the help of microorganisms has provided a cost-effective and eco-friendly strategy. Copper nanoparticles were synthesized from *Escherichia* sp. SINT7, which is copper resistant. The biogenic nanoparticles were shown to degrade azo dye and textile effluent. At a lower concentration of 25 mg/L, the reduction of reactive black-5, congo red, direct blue-1, and malachite green was 83.61, 97.07, 88.42, and 90.55%, respectively, while this was reduced at 100 mg/L concentration to 76.84, 83.90, 62.32, and 31.08%, respectively. The industrial effluent was also treated and there was a reduction in the suspended solids and chloride and phosphate ions in treated samples. The performance of such biogenic nanoparticles gives a boost to cost-effective and sustainable production from industries (Noman et al., 2020). Cheng et al. (2019) prepared iron-sulfur nanoparticles without using extra sulfur. These nanoparticles were able to degrade Napthol Green B dye through the extracellular transfer of electrons. The use of *Pseudoalteromonas* sp. CF10-13 in the preparation of nanoparticles provides an eco-friendly method for biodegradation. The endogenous production of nanoparticles inhibited the production of harmful gases and metal complexes. The use of biogenic particles is a superior technology to apply in the remediation of industrial effluents. But besides the production of nanoparticles directly from the microorganisms, there are several other ways in which microorganisms can help in boosting nanotechnology. For instance, the microorganisms could provide catalytic enzymes which, along with nanoparticles, help in remediation of effluents. Table 1 provides brief information about the use of nanotechnology in the bioremediation of wastewater. Microorganisms also help in the production of useful products from industrial waste, which will be discussed further.

**TABLE 1:** Bioremediation of different industrial effluents using advanced nanotechnology processes.

Sr. no.	Nanotechnology applied	Modification	Associated microorganisms	Removal or adsorption capacity	Advantage/Mechanism	Specific feature	References
1.	NiO and MgO nanoparticles	Silica-embedded	–	Maximum uptake of 41.36, 13.76, 7.23 (ions per nm^2^) for Cr^3+^, Cu^2+^, and Zn^2+^	Spontaneous, endothermic, and physical adsorption of Cu^2+^ and Cr^3+^ and exothermic and chemical of Zn^2+^	Regeneration and reusability proved sustainability	Abuhatab et al., 2020
2.	Electrospunnanofibrous webs	Bacterial encapsulation	*Pseudomonas aeruginosa*	55–70% removal of methylene blueat different concentrations	Biological removal of dye	Genetic engineering or more potent bacterial cell could prove more promising	Sarioglu et al., 2017
3.	Mesoporous organosilica nanoparticles (MONs)	Incorporation of ferrocene	–	High removal rate of dyes by MONs-50% and metals by MONs-25%	More surface area and πnjugation derived from non-covalent interaction facilitated by ferrocene	Novel organic-inorganic hybrid nanomaterial	Yang et al., 2019
4.	Cobalt and cobalt oxide nanoparticles	Microwave and reductive chemical heating	–	43.6 and 39.4% degradation of murexide dye by Cobalt and cobalt oxide nanoparticles, respectively	Irradiation and large surface area	Greener, easy, and faster to make, cost-effective and photocatalytic degradation efficiency	Adekunle et al., 2020
5.	Electrospuncyclodextrinfibers	Bacterial encapsulation	*Lysinibacillus* sp. NOSK	Removal efficiency of Ni(II) = 70 ± 0.2%, Cr(VI) = 58 ± 1.4% and Reactive black 5 = 82 ± 0.8	Bacterial bioremediation	Cyclodextrin provides extra carbon source for growth of bacteria	San Keskin et al., 2018
6.	Zirconia nanoparticles	Synthesis from microbial cell free culture supernatant	*Pseudomonas aeruginosa*	Tetracycline adsorption of 526.32 mg/g	Chemisorptions and strong electrostatic interaction among zwitter ions	Green synthesis of nanoparticles and sustainable bioremediation	Debnath et al., 2020
7.	Enzyme immobilized nanoparticles	Laccase immobilization	*P. ostreatus*	Degradation of bisphenol-A = 90% and carbamazepine = 10%	Oxidation by immobilized laccase	Reusable enzyme and cost-effective	Ji et al., 2017
8.	Graphene oxide and carbon nanotubes	Nano-sized nickel metal organic framework	–	Methylene blue adsorption of 222 mg/g	Hydrophobic and/or π-π interactions, high surface area, occurrence of the pores among the MOFs and the platforms and diverse morphological features of mixed nanocomposites	Superior interaction of nanocomposite	Ahsan et al., 2020
9.	Silica nanoparticles	Synthesized from actinomycetes	Actinomycetes	80% decolorization of industrial effluent	Photocatalytic degradation	Cost-effective and sustainable	Mohanraj et al., 2020

## Nanotechnology and Enzyme Technology

The combination of enzymes with nanotechnology is of the utmost importance to make nanomaterials less harmful to the environment. When enzyme molecules are present with nanomaterials, they minimize their cell interaction through steric hindrances and decrease in the surface energy (Dwevedi, 2019). Since enzymes are eco-friendly and provide a supplementary distinctiveness of catalysis, this makes nanomaterials more adaptable and efficient in bioremediation and green energy production. Conversely, immobilized enzymes on nanomaterials are highly stable due to resistance in unfolding, being less vulnerable to diffusional constraints, being able to be used in multiple cycles, and having enhanced kinetic characteristics (Ding et al., 2015). The large surface area of nanomaterials improves immobilization efficiency through elevated enzyme loading. Immobilized enzymes can be easily separated from the reaction blend, predominantly when the immobilizing matrix of magnetic nanomaterials is used. Multimeric enzymes such as oxidoreductases can also be stabilized by immobilizing them on nanomaterials. Enzyme immobilization on solid substrates leads to changes in structures, mainly increasing β-sheet structure and decreasing the α-helical structure; such modifications are not observed when nanomaterials are used for enzymes’ immobilization (Secundo, 2013).

Studies have revealed the superiority of the combination of these two technologies. Darwesh et al. (2019) showed the effect of immobilized peroxidase enzyme on wastewater bioremediation. They found that glutaraldehyde-modified iron oxide magnetic nanoparticles provided pH and temperature stable immobilized enzymes. The immobilized peroxidase enzyme was able to remove green and red azo dyes individually in 4 h. It took 6 h to completely remove the dyes when a combination of both the dyes was used at the same time at lab-scale experiments (Darwesh et al., 2019). Laccase is widely used for the treatment of industrial effluents. Various composites of magnetic nanoparticles have been utilized to immobilize laccase for biodegradation. In a study, an Fe_3_O_4_ and chitosan composite was used as a magnetic carrier for laccase immobilization. The covalently bound laccase was stable and able to remove 2, 4-Dichloro-Phenol (2, 4-DCP) and 4-Choloro-Phenol (4-CP) effectively even up to 10 cycles. The breakdown of 4-CP and 2,4-DCP reached 75.5% and 91.4% after 12 h (Zhang et al., 2020). In another experiment, Li et al. (2020) used Fe_3_O_4_ core and chelated Cu^2+^ of carbon shell to immobilize laccase. These Fe_3_O_4_@C-Cu^2+^ nanoparticles possessed a simple immobilization method, high enzyme activities, and high loading capacity, reusability, and stabilities of the immobilized laccase. The immobilized laccase was able to degrade synthetic dyes, reactive blue 19, crystal violet, Procion red MX-5B, azophloxine, brilliant green, and malachite green to approximately 81, 79, 75, 88, 93, and 99 (%) respectively in the first cycle. After 10 continuous reuses, the degradation rates were 65, 71, 60, 78, 80, and 94 (%), respectively (Li et al., 2020). Similarly, immobilized lignin peroxidase on Fe_3_O_4_@SiO_2_@polydopamine nanoparticles was able to reduce organic pollutants to a higher extent than the free enzyme. Immobilized lignin peroxidase dissipated 100% of dibutyl phthalate, phenol, tetracycline, and 5-chlorophenol. The removal of benzo(a)pyrene, phenanthrene, and fluoranthene was observed at 65, 79, and 73%, respectively (Guo et al., 2019). In another study, the recombinant cyanate hydratase was immobilized on iron-oxide-filled magnetic MWCNTs. The action of the immobilized enzyme on synthetic wastewater sample was able to remove Cu, Fe, Cr, and Pb by 29.63, 35.53, 39.31, and 34.48%, respectively. Also, the amount of cyanate was reduced by ≥ 84% (Ranjan et al., 2018, 2019). Thus, it is evident from such studies that enzyme technology, along with nanotechnology, provides a stable and efficient environment for the degradation of industrial effluents.

## Valorization of Waste Using Microorganisms and Nanotechnology

Conversion of waste materials to useful products using technology is attracting the attention of researchers around the world. Using this approach, we can reduce waste and generate useful products simultaneously. This practice is widely used in industries for the production of adsorbents, clinker, biogas, biohydrogen, biomolecules, and many more products (Gupta and Shukla, 2020). Nanotechnology has helped in the enhancement of the production rate for efficient conversion of waste into resources. Kumar and colleagues in 2019 described the use of nanoparticles to enhance dark fermentation reactions for increased biohydrogen production (Kumar et al., 2019). Supplementation of fermentative bacteria with nanoparticles has opened new avenues for biohydrogen generation from wastewater. Elreedy et al. (2019) utilized mixed culture bacteria along with single, dual, and multiple nanoparticles to generate biohydrogen. They found that biohydrogen production was the maximum (14% more than the single nanoparticles use) when multiple nanoparticles were used. The different nanoparticles increased hydrogenase and dehydrogenase activity, leading to increased biohydrogen production (Elreedy et al., 2019). Similarly, the addition of both nickel oxide and hematite nanoparticles gave 1.2–4.5-fold increased biohydrogen production than the sole nanoparticles. The highest hydrogen yield of 8.83 mmol/g COD was obtained in the combination of nanoparticles. This increase is owed to the increased activity of hydrogenase and ferredoxin oxidoreductase enzymes (Gadhe et al., 2015). Thus, nanotechnology can also be used to generate green energy for sustainable industrial growth and eco-friendly production.

## Future Perspective and Challenges

Nanotechnology has generated interest among researchers due to its beneficial effects, such as its large provided surface area, the capability of multiple uses, its stability at harsh conditions, easy and efficient manipulations in materials, increased interaction, and many more. The integration of microorganisms and enzymes with nanotechnology has provided a greener approach toward the management of industrial effluents (Dixit et al., 2020; Zhang et al., 2020). The risk associated with chemically synthesized nanoparticles can be minimized through the use of microorganisms. The residues left are either biocompatible or can be easily separated using simple filtration/precipitation techniques. The bigger challenge lies in the commercialization of these nanotechnological aspects. Only 1% of these nanotechnological aspects are commercialized so far (Dwevedi, 2019). So, the application of these easy and efficient microorganisms-assisted nanotechnology techniques on a large scale will be a stepping stone for industries. This requires continuous support and confirmation from researchers and government funding to nurture the power of nanotechnology for sustainable and cost-effective production in industries.

## Conclusion

Nanotechnology integrated with microorganisms has provided a green approach toward the bioremediation of industrial effluents. The discussed generation of nanomaterials with the help of microorganisms provides superior avenues for cost-effective and sustainable effluent remediation. Enzyme nanotechnology has provided stable, highly active, and long-lasting enzymes that offer multiple uses. This technique should be pursued further at a commercial scale to exploit its full potential. Further work can be accelerated toward the generation of biohydrogen and bioelectricity from industrial waste, as discussed in waste valorization. This will boost the industrial economy through green energy generation.

## Author Contributions

Mandeep wrote the first draft of the manuscript. The final draft was read and edited by PS. Both authors listed have made a substantial, direct and intellectual contribution to the work, and approved it for publication.

## Conflict of Interest

The authors declare that the research was conducted in the absence of any commercial or financial relationships that could be construed as a potential conflict of interest.

## References

[B1] Abuhatab S. El-Qanni A. Al-Qalaq H. Hmoudah M. Al-Zerei W. (2020). Effective adsorptive removal of Zn2+, Cu2+, and Cr3+ heavy metals from aqueous solutions using silica-based embedded with NiO and MgO nanoparticles. *J. Environ. Manag*. 268:110713. 10.1016/j.jenvman.2020.110713 32510447

[B2] Adekunle A. S. Oyekunle J. A. Durosinmi L. M. Oluwafemi O. S. Olayanju D. S. Akinola A. S. (2020). Potential of cobalt and cobalt oxide nanoparticles as nanocatalyst towards dyes degradation in wastewater. *Nano Struct. Nano Obj.* 2:100405 10.1016/j.nanoso.2019.100405

[B3] Ahsan M. A. Jabbari V. Imam M. A. Castro E. Kim H. Curry M. L. (2020). Nanoscale nickel metal organic framework decorated over graphene oxide and carbon nanotubes for water remediation. *Sci. Tot. Environ*. 69:134214. 10.1016/j.scitotenv.2019.134214 31514030

[B4] Almomani F. Bhosale R. Khraisheh M. Almomani T. (2020). Heavy metal ions removal from industrial wastewater using magnetic nanoparticles (MNP). *Appl. Surf. Sci.* 506:144924 10.1016/j.apsusc.2019.144924

[B5] Baruah A. Chaudhary V. Malik R. Tomer V. K. (2019). Nanotechnology based solutions for wastewater treatment. *Nanotechnol. Water Wastewater Treat.* 2019 337–368. 10.1016/b978-0-12-813902-8.00017-4

[B6] Bolade O. P. Williams A. B. Benson N. U. (2020). Green synthesis of iron-based nanomaterials for environmental remediation: A review. *Environ. Nanotechnol. Monit. Manag*. 13:100279 10.1016/j.enmm.2019.100279

[B7] Cheng S. Li N. Jiang L. Li Y. Xu B. Zhou W. (2019). Biodegradation of metal complex Naphthol Green B and formation of iron–sulfur nanoparticles by marine bacterium Pseudoalteromonassp CF10-13. *Bioresour. Technol*. 273 49–55. 10.1016/j.biortech.2018.10.082 30408643

[B8] Corsi I. Winther-Nielsen M. Sethi R. Punta C. Della Torre C. Libralato G. (2018). Ecofriendly nanotechnologies and nanomaterials for environmental applications: key issue and consensus recommendations for sustainable and ecosafenanoremediation. *Ecotoxicol. Environ. Saf*. 154 237–244. 10.1016/j.ecoenv.2018.02.037 29476973

[B9] Darwesh O. M. Matter I. A. Eida M. F. (2019). Development of peroxidase enzyme immobilized magnetic nanoparticles for bioremediation of textile wastewater dye. *J. Environ. Chem. Eng.* 7:102805 10.1016/j.jece.2018.11.049

[B10] Debnath B. Majumdar M. Bhowmik M. Bhowmik K. L. Debnath A. Roy D. N. (2020). The effective adsorption of tetracycline onto zirconia nanoparticles synthesized by novel microbial green technology. *J. Environ. Manag.* 261:110235. 10.1016/j.jenvman.2020.110235 32148305

[B11] Deshpande B. D. Agrawal P. S. Yenkie M. K. N. Dhoble S. J. (2020). Prospective of nanotechnology in degradation of waste water: A new challenges. *Nano Struct. Nano Obj.* 22:100442 10.1016/j.nanoso.2020.100442

[B12] Ding S. Cargill A. A. Medintz I. L. Claussen J. C. (2015). Increasing the activity of immobilized enzymes with nanoparticle conjugation. *Curr. Opi. Biotechnol.* 34 242–250. 10.1016/j.copbio.2015.04.005 25957941

[B13] Dixit M. Liu H. Luo J. Shukla P. (2020). Effluents detoxification from pulp and paper industry using microbial engineering and advanced oxidation techniques. *J. Hazard. Mater.* 398:122998. 10.1016/j.jhazmat.2020.122998 32502804

[B14] Dwevedi A. (2019). *Solutions to Environmental Problems Involving Nanotechnology and Enzyme Technology.* Cambridge, CA: Academic Press.

[B15] Egbosiuba T. C. Abdulkareem A. S. Kovo A. S. Afolabi E. A. Tijani J. O. Roos W. D. (2020). Enhanced adsorption of As (V) and Mn (VII) from industrial wastewater using multi-walled carbon nanotubes and carboxylated multi-walled carbon nanotubes. *Chemosphere* 2020:126780. 10.1016/j.chemosphere.2020.126780 32353809

[B16] Elreedy A. Fujii M. Koyama M. Nakasaki K. Tawfik A. (2019). Enhanced fermentative hydrogen production from industrial wastewater using mixed culture bacteria incorporated with iron, nickel, and zinc-based nanoparticles. *Water Res.* 151 349–361. 10.1016/j.watres.2018.12.043 30616047

[B17] Gadhe A. Sonawane S. S. Varma M. N. (2015). Influence of nickel and hematite nanoparticle powder on the production of biohydrogen from complex distillery wastewater in batch fermentation. *Int. J. Hydrogen Energ*. 40 10734–10743. 10.1016/j.ijhydene.2015.05.198

[B18] Govarthanan M. Jeon C. H. Jeon Y. H. Kwon J. H. Bae H. Kim W. (2020). Non-toxic nano approach for wastewater treatment using Chlorella vulgaris exopolysaccharides immobilized in iron-magnetic nanoparticles. *Int. J. Biol. Macromol.* 162 1241-1249. 10.1016/j.ijbiomac.2020.06.227 32599232

[B19] Guo J. Liu X. Zhang X. Wu J. Chai C. Ma D. (2019). Immobilized lignin peroxidase on Fe3O4@ SiO2@ polydopamine nanoparticles for degradation of organic pollutants. *Int. J. Biol. Macromol*. 138 433–440. 10.1016/j.ijbiomac.2019.07.105 31325503

[B20] Gupta G. K. Shukla P. (2020). Insights into the resources generation from pulp and paper industry wastes: challenges, perspectives and innovations. *Bioresour. Technol*. 297:122496. 10.1016/j.biortech.2019.122496 31831257

[B21] Hoseinian F. S. Rezai B. Kowsari E. Chinnappan A. Ramakrishna S. (2020). Synthesis and characterization of a novel nanocollector for the removal of nickel ions from synthetic wastewater using ion flotation. *Sep. Purif. Technol*. 240:116639 10.1016/j.seppur.2020.116639

[B22] Ji C. Nguyen L. N. Hou J. Hai F. I. Chen V. (2017). Direct immobilization of laccase on titania nanoparticles from crude enzyme extracts of P. ostreatus culture for micro-pollutant degradation. *Sep. Purif. Technol*. 178 215–223. 10.1016/j.seppur.2017.01.043

[B23] Kariim I. Abdulkareem A. S. Abubakre O. K. (2020). Development and characterization of MWCNTs from activated carbon as adsorbent for metronidazole and levofloxacin sorption from pharmaceutical wastewater: Kinetics, isotherms and thermodynamic studies. *Sci. Afr.* 7:e00242 10.1016/j.sciaf.2019.e00242

[B24] Kumar G. Mathimani T. Rene E. R. Pugazhendhi A. (2019). Application of nanotechnology in dark fermentation for enhanced biohydrogen production using inorganic nanoparticles. *Int. J. Hydrogen Energ.* 44 13106–13113. 10.1016/j.ijhydene.2019.03.131

[B25] Kumari P. Alam M. Siddiqi W. A. (2019). Usage of nanoparticles as adsorbents for waste water treatment: An emerging trend. *Sustain. Mater. Technol*. 22:e00128 10.1016/j.susmat.2019.e00128

[B26] Leo C. P. Chai W. K. Mohammad A. W. Qi Y. Hoedley A. F. A. Chai S. P. (2011). Phosphorus removal using nanofiltration membranes. *Water Sci. Technol*. 64 199–205. 10.2166/wst.2011.598 22053475

[B27] Li Z. Chen Z. Zhu Q. Song J. Li S. Liu X. (2020). Improved performance of immobilized laccase on Fe3O4@ C-Cu2+ nanoparticles and its application for biodegradation of dyes. *J. Hazard. Mater.* 399:123088. 10.1016/j.jhazmat.2020.123088 32937718

[B28] Liu W. Wang D. Soomro R. A. Fu F. Qiao N. Yu Y. (2019). Ceramic supported attapulgite-graphene oxide composite membrane for efficient removal of heavy metal contamination. *J. Memb. Sci*. 591:117323 10.1016/j.memsci.2019.117323

[B29] Mahanty S. Chatterjee S. Ghosh S. Tudu P. Gaine T. Bakshi M. (2020). Synergistic approach towards the sustainable management of heavy metals in wastewater using mycosynthesized iron oxide nanoparticles: Biofabrication, adsorptive dynamics and chemometric modeling study. *J. Water Proces. Eng*. 37:101426 10.1016/j.jwpe.2020.101426

[B30] Mohamed S. N. Thomas N. Tamilmani J. Boobalan T. Matheswaran M. Kalaichelvi P. (2020). Bioelectricity generation using iron (II) molybdate nanocatalyst coated anode during treatment of sugar wastewater in microbial fuel cell. *Fuel* 277:118119 10.1016/j.fuel.2020.118119

[B31] Mohammadi S. Z. Safari Z. Madady N. (2020). A novel Co3O4@ SiO2 magnetic nanoparticle-nylon 6 for high efficient elimination of Pb (II) ions from wastewater. *Appl. Surf. Sci.* 514:145873 10.1016/j.apsusc.2020.145873

[B32] Mohanraj R. Gnanamangai B. M. Poornima S. Oviyaa V. Ramesh K. Vijayalakshmi G. (2020). “Decolourisation efficiency of immobilized silica nanoparticles synthesized by actinomycetes,” in *Materials Today: Proceedings*, (Netherland: Elsevier).

[B33] Najafpoor A. Norouzian-Ostad R. Alidadi H. Rohani-Bastami T. Davoudi M. Barjasteh-Askari F. (2020). Effect of magnetic nanoparticles and silver-loaded magnetic nanoparticles on advanced wastewater treatment and disinfection. *J. Mol. Liq.* 303:112640 10.1016/j.molliq.2020.112640

[B34] Nogueira H. P. Toma S. H. Silveira A. T. Carvalho A. A. Fioroto A. M. Araki K. (2019). Efficient Cr (VI) removal from wastewater by activated carbon superparamagnetic composites. *Microchem. J*. 149:104025 10.1016/j.microc.2019.104025

[B35] Noman M. Shahid M. Ahmed T. Niazi M. B. K. Hussain S. Song F. (2020). Use of biogenic copper nanoparticles synthesized from a native *Escherichia* sp. as photocatalysts for azo dye degradation and treatment of textile effluents. *Environ. Pollut.* 257:113514. 10.1016/j.envpol.2019.113514 31706778

[B36] Palit S. Hussain C. M. (2020). *Functionalization of nanomaterials for industrial applications: recent and future perspectives. In Handbook of Functionalized Nanomaterials for Industrial Applications.* Amsterdam: Elsevier, 3–14.

[B37] Pourrahim S. Salem A. Salem S. Tavangar R. (2020). Application of solid waste of ductile cast iron industry for treatment of wastewater contaminated by reactive blue dye via appropriate nano-porous magnesium oxide. *Environ. Pollut.* 256:113454. 10.1016/j.envpol.2019.113454 31679878

[B38] Ranjan B. Pillai S. Permaul K. Singh S. (2018). A novel strategy for the efficient removal of toxic cyanate by the combinatorial use of recombinant enzymes immobilized on aminosilane modified magnetic nanoparticles. *Bioresour. Technol*. 253 105–111. 10.1016/j.biortech.2017.12.087 29331825

[B39] Ranjan B. Pillai S. Permaul K. Singh S. (2019). Simultaneous removal of heavy metals and cyanate in a wastewater sample using immobilized cyanate hydratase on magnetic-multiwall carbon nanotubes. *J. Hazard. Mater*. 363 73–80. 10.1016/j.jhazmat.2018.07.116 30308367

[B40] San Keskin N. O. Celebioglu A. Sarioglu O. F. Uyar T. Tekinay T. (2018). Encapsulation of living bacteria in electrospuncyclodextrin ultrathin fibers for bioremediation of heavy metals and reactive dye from wastewater. *Colloid. Surface. B*. 161 169–176. 10.1016/j.colsurfb.2017.10.047 29078166

[B41] Sarioglu O. F. San Keskin N. O. Celebioglu A. Tekinay T. Uyar T. (2017). Bacteria encapsulated electrospunnanofibrous webs for remediation of methylene blue dye in water. *Colloid. Surface. B* 152 245–251. 10.1016/j.colsurfb.2017.01.034 28119219

[B42] Secundo F. (2013). Conformational changes of enzymes upon immobilisation. *Chem. Soc. Rev*. 42 6250–6261. 10.1039/c3cs35495d 23482973

[B43] Shalaby M. S. Abdallah H. Cenian A. Sołowski G. Sawczak M. Shaban A. M. (2020). Laser Synthesized Gold-Nanoparticles, Blend NF Membrane for phosphate Separation from Wastewater. *Sep.Purif. Technol.* 247:116994 10.1016/j.seppur.2020.116994

[B44] Takmil F. Esmaeili H. Mousavi S. M. Hashemi S. A. (2020). Nano-magnetically modified activated carbon prepared by oak shell for treatment of wastewater containing fluoride ion. *Adv. Powder Technol.* 31 3236-3245. 10.1016/j.apt.2020.06.05

[B45] Yang S. Chen S. Fan J. Shang T. Huang D. Li G. (2019). Novel mesoporous organosilica nanoparticles with ferrocene group for efficient removal of contaminants from wastewater. *J. Colloid Interf. Sci*. 554 565–571. 10.1016/j.jcis.2019.07.037 31326788

[B46] Zhang K. Yang W. Liu Y. Zhang K. Chen Y. Yin X. (2020). Laccase immobilized on chitosan-coated Fe3O4 nanoparticles as reusable biocatalyst for degradation of chlorophenol. *J. Mol. Struct.* 1220:128769 10.1016/j.molstruc.2020.128769

